# Plasma calprotectin and its association with cardiovascular disease manifestations, obesity and the metabolic syndrome in type 2 diabetes mellitus patients

**DOI:** 10.1186/1471-2261-14-196

**Published:** 2014-12-19

**Authors:** Lise Pedersen, Mads Nybo, Mikael Kjær Poulsen, Jan Erik Henriksen, Jordi Dahl, Lars Melholt Rasmussen

**Affiliations:** Department of Clinical Biochemistry and Pharmacology, Odense University Hospital, Sdr. Boulevard 29, 5000 Odense C, Denmark; Department of Cardiology, Sygehus Lillebælt, Kabbeltoft 25, 7100 Vejle, Denmark; Department of Endocrinology, Diabetes Research Center, Odense University Hospital, Sdr. Blvd 29, 5000 Odense C, Denmark; Department of Cardiology, Odense University Hospital, Sdr. Blvd 29, 5000 Odense C, Denmark

**Keywords:** Calprotectin, MRP8/14, Cardiovascular disease, Inflammation, Type 2 diabetes, Obesity, Metabolic syndrome, Automated assay, Reference range

## Abstract

**Background:**

Plasma calprotectin is a potential biomarker of cardiovascular disease (CVD), insulin resistance (IR), and obesity. We examined the relationship between plasma calprotectin concentrations, CVD manifestations and the metabolic syndrome (MetS) in patients with type 2 diabetes mellitus (T2DM) in order to evaluate plasma calprotectin as a risk assessor of CVD in diabetic patients without known CVD.

**Methods:**

An automated immunoassay for determination of plasma calprotectin was developed based on a fecal Calprotectin ELIA, and a reference range was established from 120 healthy adults. Plasma calprotectin concentrations were measured in 305 T2DM patients without known CVD. They were screened for carotid arterial disease, peripheral arterial disease (PAD), and myocardial ischemia (MI) by means of carotid artery ultrasonography, peripheral ankle and toe systolic blood pressure measurements, and myocardial perfusion scintigraphy.

**Results:**

The reference population had a median plasma calprotectin concentration of 2437 ng/mL (2.5-97.5% reference range: 1040–4262 ng/mL). The T2DM patients had significantly higher concentrations (3754 ng/mL, p < 0.0001), and within this group plasma calprotectin was significantly higher in patients with MetS (p < 0.0001) and also in patients with autonomic neuropathy, PAD, and MI compared with patients without (p < 0.001, p = 0.021 and p = 0.043, respectively). Plasma calprotectin was by linear regression analysis found independently associated with BMI, C-reactive protein, and HDL cholesterol. However, plasma calprotectin did not predict autonomic neuropathy, PAD, MI or CVD when these variables entered the multivariable regression analysis as separate outcome variables.

**Conclusion:**

T2DM patients had higher concentrations of plasma calprotectin, which were associated with obesity, MetS status, autonomic neuropathy, PAD, and MI. However, plasma calprotectin was not an independent predictor of CVD, MI, autonomic neuropathy or PAD.

**Trial registration number:**

NCT00298844

## Background

The inflammatory myeloid-related protein complex calprotectin, also known as MRP8/14, is a heterodimer comprised of two intracellular calcium-binding proteins, S100A8 (MRP8) and S100A9 (MRP14), predominantly expressed in activated human neutrophils, monocytes and macrophages. Calprotectin is actively secreted during the stress response of phagocytes [1] and was found to be associated with inflammation more than 20 years ago [2]. Recently, calprotectin was identified as an endogenous activator of Toll-like receptor 4 and as receptor for advanced glycation end products (RAGE) [3] and calprotectin is believed to function both as an intracellular differentiation marker for phagocytes and as an extracellular protein complex (a damage-associated molecular pattern (DAMP) molecule) [4]. Elevated plasma levels of calprotectin have been reported in a variety of chronic inflammatory conditions, including rheumatoid arthritis, allograft rejection, inflammatory bowel disease, cancer and lung diseases [5].

*In vivo* studies in mice have shown that calprotectin promote atherosclerosis [6]. Furthermore, elevated calprotectin levels have been reported to predict microvascular alterations in type 2 diabetes (T2DM) patients [7] and was found to be an early and sensitive marker of acute coronary syndrome [8] and nonfatal myocardial infarction [9]. In a screening approach among healthy individuals, increased plasma concentrations of calprotectin were found to predict the risk of future cardiovascular events [10]. Furthermore, levels of plasma calprotectin appear to increase earlier than other markers of myocardial necrosis (myoglobin, creatine kinase–MB, and troponin), and high levels are associated with an increased risk of recurrent cardiovascular events [9].

T2DM is a disease characterized by increasing insulin resistance over time and is commonly associated with hypertension, hyperlipidemia, and obesity. These patients often has a number of discrete CVD risk factors that together with insulin resistance is known as the ‘metabolic syndrome’ as defined by the World Health Organization and the NCEP Adult Treatment Panel III [11]. The major death cause in T2DM patients is cardiovascular disease (CVD) [12].

Early identification and characterization of the risk of a cardiovascular event in T2DM patients is therefore important in order to prevent these events. The identification of a biomarker that can predict CVD would therefore be useful in routine clinical use. The aim of the present study was to investigate the association of plasma calprotectin levels with CVD and other complications associated with T2DM in a well-characterized T2DM cohort.

## Methods

### Control cohort for establishment of a reference range

Serum samples were obtained from a cohort of 120 adult Danish blood donors in August and September 2011 at Odense University Hospital. All 120 individuals were healthy and fulfilled the general demands for blood donation. Serum samples were obtained from 62 females and 58 males with an age ranging from 19 to 66 years. They were stratified into subgroups by gender and age (<40 years and ≥40 years). Informed consent was obtained prior to donation of blood and the study was performed according to the Declaration of Helsinki. All samples were aliquoted and stored at −80°C until analysis.

### Patient cohort

We consecutively evaluated 753 T2DM patients referred to the Diabetes Clinic at Odense University Hospital, Denmark, from January 2006 to December 2007 of which 305 patients met the inclusion criteria as previously reported [13]. Briefly, the inclusion criteria were (1) age >20 years, and (2) fasting C-peptide >250 pmol/L, while the exclusion criteria were (1) any medical history of CVD (stroke, myocardial infarction, coronary or peripheral revascularization, or ankle/toe systolic blood pressure <50/30 mmHg), (2) suspected short lifespan due to malignant disease and/or end-stage kidney disease, (3) pregnancy or planned pregnancy during the study period, (4) body weight >150 kg, or (5) physical or mental disability not enabling participation in the study.

The T2DM diagnosis was made according to the WHO criteria [14]. Patients were screened for CVD by physical examination, B-mode ultrasound scans of the carotid arteries, ankle and toe systolic blood pressure measurements and myocardial perfusion scintigraphy (MPS) as previously reported [13]. From the MPS images a summed stress score (SSS) was calculated. The blood samples collected from the T2DM patients were EDTA-plasma; all samples were centrifuged, plasma was aliquoted and stored at −80°C until analysis. The study was carried out according to Good Clinical Practice, followed the Helsinki II Declaration, was approved by the Local Ethics Committee (De Videnskabsetiske Komitéer for Region Syddanmark), and is registered at http://www.clinicaltrials.gov (Identification-nr: NCT00298844). All participants gave written, informed consent.

### Definition of obesity, insulin resistance and metabolic syndrome

Overweight and obesity were defined as a body mass index (BMI) 25–30 kg/m^2^ and > 30 kg/m^2^, respectively, as proposed by WHO. Insulin resistance (IR) was calculated using a homeostasis model assessment (HOMA) of IR (HOMA-IR = fasting glucose (mmol/L) × fasting insulin (μU/mL)/22.5) [15]. The presence of the metabolic syndrome was assessed according to the American Heart Association guidelines, defined as waist circumference ≥102 cm in men and ≥88 cm in women, triglycerides ≥1.69 mmol/L, HDL-Cholesterol <1.03 mmol/L in men and <1.29 mmol/L in women, blood pressure ≥130/85 mmHg, and fasting plasma glucose ≥5.6 mmol/L [16].

### Laboratory analyses

#### Calprotectin measurement

Analysis of serum/plasma calprotectin was performed with a new fully automated Fluorescent Enzyme Sandwich Immuno Assay designed for measurement of fecal Calprotectin (ELIA Calprotectin for ImmunoCap250, 250-5611-01/UK). The ELIA calprotectin assay uses recombinant human calprotectin as standard and the wells are coated with monoclonal antibodies directed towards calprotectin. After incubation with the sample the wells were washed to remove non-bound calprotectin, and enzyme-labeled antibodies against human calprotectin were added to form a calprotectin-conjugate complex. After incubation an additional wash was performed in order to remove non-bound conjugate and finally, development solution was added. After terminating the reaction fluorescence was measured directly using an ImmunoCap250 (Thermo Scientific Life Technologies, Waltham, MA, USA). Data from all measurements were plotted as a four-parameter logistic (4PL) curve fit of the fluorescent counts versus standard concentrations using GraphPad Prism software (version 5; GraphPad Software, La Jolla, CA) from which the concentrations of plasma calprotectin were calculated. The ELIA Fecal Calprotectin assay was validated for use on plasma/serum samples. Prior to analysis, serum/plasma samples were diluted 1:100 in ELIA Sample Diluent. The linear range was determined using a sample with a calprotectin concentration of 14700 ng/mL, which was serially diluted to create ten samples that were tested in duplicate. The results were linear over a concentration range of 2.3-14700 ng/mL (y = 1.08× + 0.12, R^2^ = 0.997). The quantification limit of the assay was 3.0 ng/mL defined as the value 10 standard deviations (SD) above the mean value of the zero standard (n = 7). Within-run imprecision was 4.5% and between-run imprecision was 5.6% for a serum pool with a mean calprotectin concentration of 5234 ng/mL.

Since the available test material were limited to serum from the control cohort and plasma from the patient cohort, a comparison of the calprotectin levels in serum and plasma was performed:Calprotectin was measured in 58 matching plasma and serum samples, which showed a significant correlation between the two sample types (Spearman’s correlation coefficient; *r* = 0.899, *p* < 0.001) and with no statistical significant differences in the median values for the two sample types when compared by Mann–Whitney U-test (p = 0.191).

#### Other biochemical measurements

HbA1c (glycated hemoglobin) was measured by cation-exchange chromatography using Tosoh G7 (Medinor, Broendby, Denmark) with dedicated reagents. Glucose, total cholesterol, LDL cholesterol, HDL cholesterol, and high-sensitive CRP (hs-CRP) were all analyzed on a Modular Analytics P (Roche Diagnostics, Switzerland) with the methods applied as recommended by the supplier.

### Statistical analyses

Continuous variables are presented as mean and standard deviations, categorical variables as numbers and percentages with 95% confidence intervals (CI). Student’s t-test was used to test differences between independent continuous variables. Due to a non-Gaussian distribution of hs-CRP, HOMA-IR and plasma calprotectin, these parameters are presented as median and interquartile range, and the Mann–Whitney test was used to compare groups. For comparison of categorical variables with more than two groups, a Kruskall-Wallis nonparametric test was used. Due to the non-Gaussian distribution, bivariate Spearman correlation coefficients were used to describe the association between plasma calprotectin levels and other continuous variables. Multiple stepwise regression analysis was performed with plasma calprotectin concentrations as dependent variable and by entering independent variables with the highest partial correlation coefficient at each step with an F-value probability for inclusion of 0.05 and 0.01 for removal.

Logistic regression analysis was performed using autonomic neuropathy, PAD and MI as separate outcome variables. The co-variables were adjusted for age, CRP and BMI. Results are reported as odds ratio (OR) with 95% CI and p-values. A p-value < 0.05 was considered statistically significant. SPSS for Windows version 20 (SPSS Inc., Chicago, Illinois) was used for calculations.

## Results

### Establishment of a reference range

The one-sample Shapiro-Wilk test of normality showed that the distribution of serum calprotectin was non-Gaussian (Z = 0.114, p = 0.001) and positively skewed (skewness = 1.256). Therefore, construction of the reference range employed a non-parametric approach. The median calprotectin concentration was 2437 ng/mL with the reference range determined as 1040–4262 ng/mL (2.5th-97.5th percentile). No significant difference between the genders were found (Mann–Whitney U-test; p = 0.805).

### Baseline characteristics and plasma calprotectin concentrations in the type 2 diabetes cohort

Baseline clinical and metabolic characteristics and the prevalence of CVD in the 305 T2DM patients have previously been reported [13, 17]. Added information from this study concerning prevalence of MetS, HOMA-IR and calprotectin concentrations are presented in Table 1.

**Table 1:** **Data are median (interquartile range) or n (%)**

	Total (n = 305)	CVD by examination (n = 183)	No CVD by examination (n = 122)
Metabolic syndrome/no metabolic syndrome	243/62	154/29	91/31
Calprotectin (ng/mL)	3754 (2288–5797)	3754 (2290–6194)	3753 (2278–5170)
HOMA-IR	3.9 (2.3-7.4)	4.4 (2.5-8.2)	3.4 (2.1-5.0)

Data are median (interquartile range) or n (%). CVD by examination = cardiovascular disease obtained from B-mode ultrasound scans of the carotid arteries, ankle and toe systolic blood pressure measurements and/or myocardial perfusion scintigraphy.

The 305 T2DM patients were obese with BMI 32.2 ± 5.8 kg/m^2^ (mean ± SD) and 243 of the 305 patients had metabolic syndrome according to the criteria of the American Heart Association. Median plasma calprotectin concentration was 3754 (2288–5797) ng/mL (median (IQR)), which was significantly higher than in the healthy reference population (Mann–Whitney U-test; p < 0.001).

The median HOMA-IR was significantly higher in T2DM patients with CVD compared to those without (4.4 (2.5-8.3) vs. 3.4 (2.1-5.5), p = 0.004), but patients with and without CVD did not differ in median plasma calprotectin concentrations (3754 (2290–6194) ng/mL vs. 3753 (2278–5170) ng/mL; median (IQR), p = 0.39). However, the median plasma calprotectin concentration was significantly higher in subjects with metabolic syndrome compared with subjects without (3916 (2468–6245) vs. 2943 (1793–4115) ng/mL), p < 0.0001).

### Correlation of plasma calprotectin with clinical, anthropometric and biochemical parameters

Partial Spearman correlation analysis of data from all 305 T2DM patients showed that plasma calprotectin levels were highly correlated with BMI, weight, waist circumference, hip circumference, age (inversely), hs-CRP, fasting plasma insulin, plasma HDL (inversely), HOMA-IR and Summed Stress Score (SSS) (all p < 0.001), while weakly correlated with fasting plasma C-peptide, diabetes duration (inversely), carotid intima media thickness (CIMT) (inversely) and triglycerides (all p < 0.05). Partial Spearman correlation analysis was also performed in T2DM patients with and without metabolic syndrome, respectively. Data are presented in Table 2.

**Table 2:** **Bivariate Spearman correlation between calprotectin and other biochemical markers and metabolic features in patients with and without metabolic syndrome**

	Metabolic syndrome (n = 243)		No metabolic syndrome (n = 62)	
	ρ	*P*	ρ	P
Age (years)	**−0.179**	**0.005**	−0.138	0.294
Weight (kg)	**0.199**	**0.002**	**0.317**	**0.014**
Height (m)	**−0.129**	**0.046**	−0.211	0.106
BMI (kg/m^2^)	**0.252**	**<0.001**	**0.446**	**<0.001**
Waist circumference (cm)	**0.237**	**0.001**	**0.257**	**0.047**
Hip circumference (cm)	**0.230**	**<0.001**	**0.285**	**0.027**
Diabetes duration (years)	**−0.172**	**0.008**	0.092	0.486
CIMT (mm)	−0.114	0.079	−0.168	0.200
Fasting p-glucose (mmol/L)	**−0.148**	**0.021**	−0.069	0.601
HbA1c (%)	−0.037	0.564	−0.052	0.691
Fasting C-peptide (pmol/L)	0.081	0.212	0.177	0.176
Fasting insulin (pmol/L)	**0.169**	**0.009**	0.247	0.057
Total cholesterol conc. (mmol/L)	−0.072	0.268	0.084	0.523
LDL-cholesterol conc. (mmol/L)	0.002	0.978	0.131	0.318
HDL-cholesterol conc. (mmol/L)	**−0.151**	**0.019**	**−0.259**	**0.045**
Triglycerides (mmol/L)	0.018	0.779	**0.289**	**0.025**
Creatinine (μmol/L)	0.000	0.995	0.022	0.868
hs-CRP (mg/L)	**0.415**	**<0.001**	**0.346**	**0.007**
HOMA-IR	0.110	0.088	0.169	0.197
SSS	**0.136**	**0.035**	0.222	0.088

Statistical significant results are shown in **bold.**

All parameters significantly correlated with plasma calprotectin in the 305 T2DM patients entered a stepwise multiple regression analysis. The independent predictors of plasma calprotectin concentrations obtained from this analysis are presented in Table 3; the ANOVA inflation factors excluded multicollinearity for these variables.

**Figure 1:**
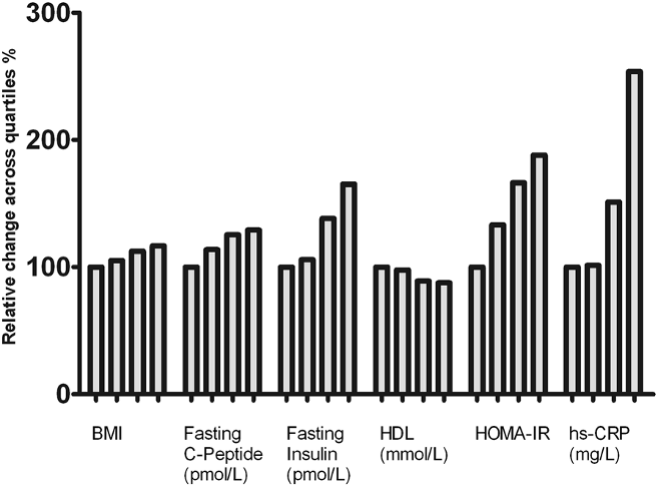
**Relative increase/decrease of BMI, fasting C-peptide, fasting insulin, HDL cholesterol, HOMA-IR and hs-CRP across calprotectin quartiles.** The difference across quartiles were significant at the p < 0.0001 (ANOVA) level for BMI and hs-CRP and at the p < 0.05 level (ANOVA) for fasting C-peptide, fasting insulin, HDL and HOMA-IR (ANOVA).

When stratifying by quartiles of calprotectin concentrations, the variables BMI, fasting C-peptide, fasting insulin, HOMA-IR and hs-CRP increased significantly across calprotectin quartiles (all p < 0.05, ANOVA), while HDL cholesterol concentrations decreased significantly across calprotectin quartiles (p < 0.001, ANOVA) (Figure 1).

**Table 3:** **Overall multiple regression analysis between plasma calprotectin levels (dependent variable) and the variables significantly correlated to this parameter in all subjects**

Independent variable	All cases(n = 305)	
Model I	β	*P*
Age (years)	−0.039	0.516
Weight (kg)	−0.249	0.016
BMI (kg/m^2^)	**0.228**	**<0.001**
Waist circumference (cm)	−0.087	0.357
Hip circumference (cm)	0.058	0.588
Diabetes duration (years)	−0.086	0.120
CIMT (mm)	−0.073	0.189
Fasting C-peptide (pmol/L)	0.030	0.617
Fasting insulin (pmol/L)	0.053	0.378
HDL-cholesterol conc. (mmol/L)	**−0.153**	**0.005**
Triglycerides (mmol/L)	−0.040	0.501
HOMA-IR	−0.004	0.943
SSS	0.015	0.788
hs-CRP (mg/L)	**0.235**	**<0.001**

Statistical significant results are shown in **bold**. Model summary: R = 0.408. Predictors; BMI, p = 0.001, hs-CRP, p < 0.001 and HDL, p = 0.005.

### Associations of calprotectin with cardiovascular disease manifestations in T2DM

**Figure 2:**
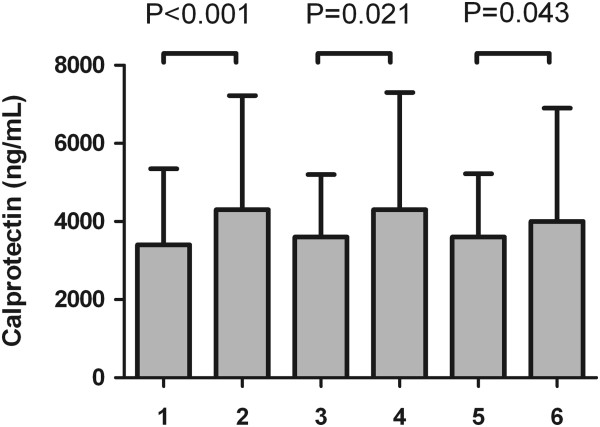
**Plasma calprotectin levels in patients without (1) or with PAD (2), without (3) or with autonomic neuropathy (4) and without (5) or with MI (6).** P-values are from a Mann–Whitney U-test.

Association of calprotectin concentrations with CVD manifestations is presented in Figure 2. Median plasma calprotectin concentrations did not differ between patients with and without CVD manifestations (p = 0.39), but were significantly higher in patients with PAD compared to patients without (4800 (900–11700) vs. 3600 (1000–12800) ng/mL, p = 0.021). There were statistical significantly higher levels of calprotectin in autonomic neuropathy compared to no autonomic neuropathy (4820 (1414–13958) vs. 3565 (902–10811) ng/mL, p < 0.001). For MI, plasma calprotectin concentrations only just reached significance higher values compared to patients without MI (4003 (1019–13187) vs. 3678 (955–12745) ng/mL, p = 0.043).Plasma calprotectin was also associated with autonomic neuropathy and with myocardial ischemia (Figure 2).

To further test the association of plasma calprotectin concentrations with disease manifestations a multivariate logistic regression analysis were performed with autonomic neuropathy, PAD and MI, as separate outcome variables. In this analysis plasma calprotectin did not show any significant association with autonomic neuropathy (adjusted OR 1.00; 95% CI: 0.958-1.045, p = 0.991), PAD (adjusted OR: 1.00; 95% CI: 1.00-1.00, p = 0.052), MI (adjusted OR: 1.00; 95% CI: 1.00-1.00, p = 0.413) nor CVD (adjusted OR: 1.00; 95% CI: 1.00-1.00, p = 0.857).

## Discussion

The major findings in our present study were (1) higher levels of plasma calprotectin in T2DM patients compared with a general population, (2) plasma calprotectin levels correlated with BMI, triglyceride, HDL cholesterol (inversely), hs-CRP, insulin and C-peptide levels as well as HOMA-IR in T2DM patients, (3) plasma calprotectin was independently associated with BMI, hs-CRP and HDL cholesterol (inversely), (4) higher levels of plasma calprotectin were found in patients with MetS compared to patients without, and (5) the plasma level of calprotectin was not associated with CVD in T2DM patients but higher levels of plasma calprotectin were found in patients with autonomic neuropathy, PAD and myocardial ischemia compared with patients without. Of note, plasma calprotectin could not predict autonomic neuropathy, PAD, myocardial ischemia or CVD in T2DM patients in a multivariate logistic regression analysis. Overall, we will try to discuss these findings and their possible clinical relevance.

The finding of higher plasma calprotectin levels in T2DM patients compared to a reference population is likely to reflect that calprotectin is released by macrophages and therefore are involved in systemic inflammatory diseases such as juvenile idiopathic arthritis [18] or sepsis [19]. T2DM is often characterized as a state of chronic, low-grade inflammation that contributes to insulin resistance and is associated with prolonged obesity and dyslipidemia [20]. Adipose tissue macrophage infiltration increases in mice and humans as they become obese [21] and abnormal fat accumulation is associated with inflammatory changes, including recruitment of macrophages and activation of endothelial cells [22]. High levels of plasma calprotectin have previously been shown to correlate with obesity in humans [19, 23, 24] and it is plausible that the higher levels of plasma calprotectin in obesity is caused by the adipose tissue macrophages and that the obesity-induced inflammation is mediated by the calprotectin/TLR-4/inflammation pathway leading to a systemically rise in plasma levels. Recently, MRP8 mRNA was found over-expressed in mouse white adipose tissue and 3 T3-L1 adipocytes suggesting that inflamed adipose tissue might be a major source of the calprotectin found in plasma [25]. *In vitro* studies by Catalan and co-workers showed a higher MRP8 mRNA expression levels in the stromovascular fraction cells in visceral adipose tissue (VAT) and they suggest a potential role of calprotectin as a chemotactic factor in the recruitment of macrophages to VAT, increasing inflammation and the development of obesity-associated co-morbidities [25]. That the expression level of MRP8 in human adipocyte culture increases upon stimulation with the cytokine TNF-alpha suggests a causative link [23].

So far, few studies have addressed the relationship between circulating levels of calprotectin, glucose homeostasis and insulin resistance. Our data indicate that the elevated levels of calprotectin in T2DM patients are linked to obesity independent of dysglycemia and insulin resistance. Mortensen and co-workers likewise found no significant association between circulating calprotectin and parameters of glucose homeostasis [24], which was also observed in a Chinese study [26]. On the other hand, Ortega and co-workers recently found plasma and urinary levels of calprotectin associated with insulin resistance and low-grade inflammation independent of obesity in T2DM patients, which lead to the hypothesis that calprotectin may be a biomarker of decreased insulin sensitivity beyond a common trait of obesity and inflammation [27]. The lack of association between calprotectin levels and glucose homeostasis in this study seems, however, to confirm that the regulation of calprotectin is independent of glucose metabolism.

Plasma calprotectin has previously been shown to be a useful biomarker of CVD risk [8–10], but the pathophysiological connection between higher plasma calprotectin concentrations and CVD is not known. It is unclear whether calprotectin is simply a marker of systemic low-grade inflammation or plays a more specific role in the processes that promotes atherosclerosis leading to CVD. Over-expression of calprotectin in the infarcted myocardium and higher levels of plasma calprotectin in patients with acute myocardial infarction seems to support a direct role of the protein complex in the pathophysiology of acute myocardial infarction [28]. Supporting this are studies that show an infiltration of monocytes expressing calprotectin in atherosclerotic lesions [29] and a higher expression of calprotectin in atherosclerotic lesions [6]. Also, calprotectin-deficient mice seem protected against vascular injury and have smaller atherosclerotic lesions, less plaque inflammation and decreased re-stenotic response [6]. The usefulness of calprotectin as a CVD predictor is, however, not straightforward: Peng and co-workers reported that diabetic patients with coronary artery disease (CAD) had elevated plasma calprotectin levels, and that the level of plasma calprotectin correlated with the severity of CAD and CIMT in patients without clinically overt CAD [26]. On the contrary, Bauman and co-workers failed to detect any significant differences in circulating calprotectin levels in patients with or without stable CAD [30]. Although this study shows slightly higher levels of plasma calprotectin in patients with MI, the lack of association in our multivariate regression analysis and the lack of consistency in cohort studies with CVD end points still indicate that the use of calprotectin as a CVD risk marker seems dubious.

In this study calprotectin correlated significantly with hs-CRP levels and when included in our multiple regression analysis hs-CRP (along with BMI and HDL) remained associated with the plasma calprotectin concentration supporting a role of calprotectin as an obesity-associated inflammation marker.

Although we not found an association of calprotectin with CVD, measurement of plasma calprotectin could still valuable when monitoring CVD or as a marker of therapy response. A recent systematic review by Micha et al. examined CVD risk and the use of the anti-inflammatory drug methotrexate and concluded that methotrexate use was associated with a 21% lower risk of CVD and 18% lower risk of myocardial infarction [31]. This lends promise to the notion that using markers of inflammation such as calprotectin may allow for interventions to decrease overall cardiovascular risk. Another well-known inflammation marker is hs-CRP that in multiple prospective studies has been shown to predict incident myocardial infarction, stroke, peripheral arterial disease, sudden cardiac death and also adds prognostic information at all levels of calculated Framingham Risk [32]. This indicates that valuable information could be retrieved using calprotectin as a marker beyond what would be obtained with hs-CRP. The availability of a fully automated plasma calprotectin assay opens up for future studies that can evaluate whether calprotectin can be used in monitoring disease progression and/or in intervention studies.

## Conclusion

In conclusion, plasma calprotectin levels were associated with the presence of MI, PAD and autonomic neuropathy in a consecutive series of T2DM patients without known CVD referred to a diabetes clinic for the first time. When adjusting for hs-CRP, BMI and HDL cholesterol those associations, however, disappeared. Also, plasma calprotectin was not a marker of insulin resistance independent of inflammation and BMI. The finding of elevated plasma calprotectin in subjects with MetS supports a role of calprotectin as a marker of obesity-associated chronic low-grade inflammation.

## Abbreviations

CVD: Cardiovascular disease
IR: Insulin resistance
MetS: Metabolic syndrome
T2DM: Type 2 diabetes mellitus
CAD: Coronary artery disease
PAD: Peripheral arterial disease
MI: Myocardial ischemia
BMI: Body mass index
HDL: High-density lipoprotein
MRP8/14: Myeloid-related protein 8/14
RAGE: Receptor for advanced glycation end products
DAMP: Damage-associated molecular pattern
MAPK: Mitogen-activated protein kinase
BP: Blood pressure
WHO: World Health Organization
MPS: Myocardial perfusion scintigraphy
SSS: Summed stress score
HOMA-IR: Homeostasis model assessment of insulin resistance
IQR: Interquartile range
CIMT: Carotid intima media thickness
TLR: Toll-like receptor.

## References

[1] Hessian PA, Edgeworth J, Hogg N (1993). MRP-8 and MRP-14, two abundant Ca(2+)-binding proteins of neutrophils and monocytes. J Leukoc Biol.

[2] Sorg C (1992). The calcium binding proteins MRP8 and MRP14 in acute and chronic inflammation. Behring Inst Mitt.

[3] Leclerc E, Fritz G, Vetter SW, Heizmann CW (2009). Binding of S100 proteins to RAGE: an update. Biochim Biophys Acta.

[4] Ehrchen JM, Sunderkotter C, Foell D, Vogl T, Roth J (2009). The endogenous Toll-like receptor 4 agonist S100A8/S100A9 (calprotectin) as innate amplifier of infection, autoimmunity, and cancer. J Leukoc Biol.

[5] Bjarnason I, Sherwood R (2001). Fecal calprotectin: a significant step in the noninvasive assessment of intestinal inflammation. J Pediatr Gastroenterol Nutr.

[6] Croce K, Gao H, Wang Y, Mooroka T, Sakuma M, Shi C, Sukhova GK, Packard RR, Hogg N, Libby P, Simon DI (2009). Myeloid-related protein-8/14 is critical for the biological response to vascular injury. Circulation.

[7] Burkhardt K, Schwarz S, Pan C, Stelter F, Kotliar K, Von EM, Sollinger D, Lanzl I, Heemann U, Baumann M (2009). Myeloid-related protein 8/14 complex describes microcirculatory alterations in patients with type 2 diabetes and nephropathy. Cardiovasc Diabetol.

[8] Altwegg LA, Neidhart M, Hersberger M, Muller S, Eberli FR, Corti R, Roffi M, Sutsch G, Gay S, Von EA, Wischnewsky MB, Luscher TF, Maier W (2007). Myeloid-related protein 8/14 complex is released by monocytes and granulocytes at the site of coronary occlusion: a novel, early, and sensitive marker of acute coronary syndromes. Eur Heart J.

[9] Morrow DA, Wang Y, Croce K, Sakuma M, Sabatine MS, Gao H, Pradhan AD, Healy AM, Buros J, McCabe CH, Libby P, Cannon CP, Braunwald E, Simon DI (2008). Myeloid-related protein 8/14 and the risk of cardiovascular death or myocardial infarction after an acute coronary syndrome in the pravastatin or atorvastatin evaluation and infection therapy: thrombolysis in myocardial infarction (PROVE IT-TIMI 22) trial. Am Heart J.

[10] Healy AM, Pickard MD, Pradhan AD, Wang Y, Chen Z, Croce K, Sakuma M, Shi C, Zago AC, Garasic J, Damokosh AI, Dowie TL, Poisson L, Lillie J, Libby P, Ridker PM, Simon DI (2006). Platelet expression profiling and clinical validation of myeloid-related protein-14 as a novel determinant of cardiovascular events. Circulation.

[11] Kannel WB, McGee DL (1979). Diabetes and cardiovascular risk factors: the Framingham study. Circulation.

[12] Morrish NJ, Wang SL, Stevens LK, Fuller JH, Keen H (2001). Mortality and causes of death in the WHO multinational study of vascular disease in diabetes. Diabetologia.

[13] Poulsen MK, Henriksen JE, Dahl J, Johansen A, Moller JE, Gerke O, Vach W, Haghfelt T, Beck-Nielsen H, Hoilund-Carlsen PF (2009). Myocardial ischemia, carotid, and peripheral arterial disease and their interrelationship in type 2 diabetes patients. J Nucl Cardiol.

[14] Alberti KG, Zimmet PZ (1998). Definition, diagnosis and classification of diabetes mellitus and its complications. Part 1: diagnosis and classification of diabetes mellitus provisional report of a WHO consultation. Diabet Med.

[15] Matthews DR, Hosker JP, Rudenski AS, Naylor BA, Treacher DF, Turner RC (1985). Homeostasis model assessment: insulin resistance and beta-cell function from fasting plasma glucose and insulin concentrations in man. Diabetologia.

[16] Grundy SM, Brewer HB, Cleeman JI, Smith SC, Lenfant C (2004). Definition of metabolic syndrome: report of the national heart, lung, and blood institute/American heart association conference on scientific issues related to definition. Circulation.

[17] Poulsen MK, Nybo M, Dahl J, Hosbond S, Poulsen TS, Johansen A, Hoilund-Carlsen PF, Beck-Nielsen H, Rasmussen LM, Henriksen JE (2011). Plasma osteoprotegerin is related to carotid and peripheral arterial disease, but not to myocardial ischemia in type 2 diabetes mellitus. Cardiovasc Diabetol.

[18] zur Schulze WA, Foell D, Frosch M, Vogl T, Sorg C, Roth J (2004). Myeloid related proteins MRP8/MRP14 may predict disease flares in juvenile idiopathic arthritis. Clin Exp Rheumatol.

[19] Nijhuis J, Rensen SS, Slaats Y, van Dielen FM, Buurman WA, Greve JW (2009). Neutrophil activation in morbid obesity, chronic activation of acute inflammation. Obesity (Silver Spring).

[20] Greenberg AS, Obin MS (2006). Obesity and the role of adipose tissue in inflammation and metabolism. Am J Clin Nutr.

[21] Weisberg SP, McCann D, Desai M, Rosenbaum M, Leibel RL, Ferrante AW (2003). Obesity is associated with macrophage accumulation in adipose tissue. J Clin Invest.

[22] Sekimoto R, Kishida K, Nakatsuji H, Nakagawa T, Funahashi T, Shimomura I (2012). High circulating levels of S100A8/A9 complex (calprotectin) in male Japanese with abdominal adiposity and dysregulated expression of S100A8 and S100A9 in adipose tissues of obese mice. Biochem Biophys Res Commun.

[23] Catalan V, Gomez-Ambrosi J, Rodriguez A, Ramirez B, Rotellar F, Valenti V, Silva C, Gil MJ, Fernandez-Real JM, Salvador J (2011). Increased levels of calprotectin in obesity are related to macrophage content: impact on inflammation and effect of weight loss. Mol Med.

[24] Mortensen OH, Nielsen AR, Erikstrup C, Plomgaard P, Fischer CP, Krogh-Madsen R, Lindegaard B, Petersen AM, Taudorf S, Pedersen BK (2009). Calprotectin–a novel marker of obesity. PLoS One.

[25] Hirata A, Kishida K, Nakatsuji H, Hiuge-Shimizu A, Funahashi T, Shimomura I (2012). High serum S100A8/A9 levels and high cardiovascular complication rate in type 2 diabetics with ultrasonographic low carotid plaque density. Diabetes Res Clin Pract.

[26] Peng WH, Jian WX, Li HL, Hou L, Wei YD, Li WM, Xu YW (2011). Increased serum myeloid-related protein 8/14 level is associated with atherosclerosis in type 2 diabetic patients. Cardiovasc Diabetol.

[27] Ortega FJ, Sabater M, Moreno-Navarrete JM, Pueyo N, Botas P, Delgado E, Ricart W, Fruhbeck G, Fernandez-Real JM (2012). Serum and urinary concentrations of calprotectin as markers of insulin resistance and type 2 diabetes. Eur J Endocrinol.

[28] Cinti S, Mitchell G, Barbatelli G, Murano I, Ceresi E, Faloia E, Wang S, Fortier M, Greenberg AS, Obin MS (2005). Adipocyte death defines macrophage localization and function in adipose tissue of obese mice and humans. J Lipid Res.

[29] Eue I, Langer C, Eckardstein A, Sorg C (2000). Myeloid related protein (MRP) 14 expressing monocytes infiltrate atherosclerotic lesions of ApoE null mice. Atherosclerosis.

[30] Baumann M, Schmaderer C, Burkhardt K, Haller B, Heemann U, Dugi K, Von EM (2011). MRP8/14 is associated with systemic inflammation in stable coronary atherosclerosis in men. Eur J Clin Investig.

[31] Micha R, Imamura F, von Wyler BM, Solomon DH, Hernan MA, Ridker PM, Mozaffarian D (2011). Systematic review and meta-analysis of methotrexate use and risk of cardiovascular disease. Am J Cardiol.

[32] Kannel WB, McGee DL (1979). Diabetes and cardiovascular disease. The framingham study. JAMA.

[CR33] The pre-publication history for this paper can be accessed here: http://www.biomedcentral.com/1471-2261/14/196/prepub

